# Potential Uses of Blockchain Technology for Outcomes Research on Opioids

**DOI:** 10.2196/16293

**Published:** 2021-08-27

**Authors:** Aldren Gonzales, Scott R Smith, Prashila Dullabh, Lauren Hovey, Krysta Heaney-Huls, Meagan Robichaud, Roger Boodoo

**Affiliations:** 1 US Department of Health and Human Services Office of the Assistant Secretary for Planning and Evaluation Office of Health Policy Washington, DC United States; 2 NORC at the University of Chicago Chicago, IL United States; 3 Department of Defense Defense Health Agency United States Navy Falls Church, VA United States

**Keywords:** blockchain, distributed ledger, opioid crisis, outcomes research, patient-centered outcomes research, mobile phone

## Abstract

The scale and severity of the opioid epidemic call for innovative, multipronged solutions. Research and development is key to accelerate the discovery and evaluation of interventions that support pain and substance use disorder management. In parallel, the use and integration of blockchain technology within research networks holds the potential to address some of the unique challenges facing opioid research. This paper discusses the applications of blockchain technology and illustrates potential ways in which it could be applied to strengthen the validity of outcomes research on the opioid epidemic. We reviewed published and gray literature to identify useful applications of blockchain, specifically those that address the challenges faced by opioid research networks and programs. We then convened a panel of experts to discuss the strengths, limitations, and feasibility of each application. Blockchain has the potential to address some of the issues surrounding health data management, including data availability, data sharing and interoperability, and privacy and security. We identified five primary applications of blockchain to opioids: clinical trials and pharmaceutical research, incentivizing data donation and behavior change, secure exchange and management of e-prescriptions, supply chain management, and secondary use of clinical data for research and public health surveillance. The published literature was limited, leading us to rely on gray literature, which was also limited in its discussion of the technical aspects of implementation. The technical expert panel provided additional context and an assessment of feasibility that was lacking in the literature. Research on opioid use and misuse is challenging because of disparate data stored across different systems, data and system interoperability issues, and legal requirements. These areas must be navigated to make data accessible, timely, and useful to researchers. Blockchain technologies have the potential to act as a facilitator in this process, offering a more efficient, secure, and privacy-preserving solution for data exchange. Among the 5 primary applications, we found that clinical trial research, supply chain management, and secondary use of data had the most examples in practice and the potential effectiveness of blockchain. More discussions and studies should focus on addressing technical questions concerning scalability and tackling practical concerns such as cost, standards, and governance around the implementation of blockchain in health care. Policy concerns related to balancing the need for data accessibility that also protects patient privacy and autonomy in revoking consent should also be examined.

## Background

### The Opioids Problem

Prevalent misuse and overdose related to prescription opioids have created a public health crisis in the United States [1]. In 2017, approximately 11.4 million people misused prescription opioids [2]. In addition, the rise of heroin use and the increase in the availability of illicit, highly potent synthetic opioids have fueled the crisis [1]. The urgency of the problem has become more evident with the increasing number of drug overdose deaths in the country. In 2018 alone, approximately 70% of more than 67,000 drug overdose deaths recorded involved opioids. Approximately 67% or 2 of 3 opioid-related overdose deaths involved synthetic opioids [3]. The Centers for Disease Control and Prevention also estimates that, on average, 130 Americans die every day from opioid overdose [4].

Although preliminary data from 2017 to 2018 showed a 2.8% decrease in opioid overdose deaths, there is broad recognition that the crisis is far from over and continued attention and additional research are needed [5]. Responding to the opioid epidemic will require innovative multidimensional solutions coordinated across sectors that take advantage of emerging technologies. The use of existing health data has been recognized as a key component in addressing the opioid epidemic. These data have the potential to support research that advances current knowledge about pain and addiction and leads to the discovery of new treatment options and interventions [6,7]. However, research efforts and interventions that target opioid use disorder are challenged by gaps in data and information exchange across sectors, causing a real obstacle to addressing the epidemic. Although several solutions are being implemented, technology is still considered an underused asset to address the crisis [8].

Specific to the opioid epidemic, different innovative health information technology (IT) solutions have been developed and implemented. The prescription drug monitoring program (PDMP) system is one tool that state governments have invested in to provide prescribers and pharmacists access to critical information regarding patients’ controlled substance prescription history. Other examples of health IT tools being implemented to support opioid-related management include the expansion of e-prescriptions [9], clinical decision support tools in electronic health record (EHR) systems [10], telehealth for addiction treatment services [11], and smartphone apps to support recovery [12].

### Blockchain

Blockchain is another rapidly evolving technology with potential applications to the opioid crisis and health system–level research issues related to data availability, interoperability, and privacy and security [13]. Blockchain is a type of distributed ledger technology that uses a peer-to-peer network to provide “a shared, immutable, and transparent append-only register of all the actions that have happened to all the participants [called “nodes,” which can be any organization or an individual who take part in the digital business transaction] of the network” [14,15].

Traditionally, records are managed and verified by a central authority. With blockchain, recording is decentralized, allowing all authorized users to keep an identical copy of transactions (also called *blocks*). To illustrate how it works, consider a spreadsheet document containing transactions that are duplicated and stored in a network of authorized computers (*nodes*) and are updated periodically. When a new transaction is made, the spreadsheet needs to be updated. This new transaction will be represented as a *block* on the web and will be distributed to authorized computers for verification. If the whole network says that the transaction is valid, that *block* will be appended to the chain and all the copies of the spreadsheet stored in the network will be reconciled with the new permanent record. This is the concept of a blockchain.

The technical details of blockchain technology are beyond the scope of this paper. However, to provide a backbone for discussing the use cases, the blockchain’s core features and multiple advantages over traditional distributed or centralized databases are summarized here. First, each block in the chain is connected using cryptography, which prevents tampering and malicious attacks. Second, the blockchain provides a full transaction history as part of its data *blocks*. This allows each node to validate the accuracy of transaction data and reach a consensus before adding another block to the blockchain, safeguarding transparency and reliability. Third, the system architecture is distributed across members of the network, allowing members to maintain control of their data while still contributing information to the collective. Fourth, because all data and transactions (eg, entering new information and updating or deleting a record) are available to those authorized in the network, blockchain brings about trust and transparency among the participants to the records [16,17].

There are two categories of blockchain systems: public and private. A public blockchain has several member nodes connected in a decentralized way that allows anyone to participate, read, and write data to the chain. As the network is public, nodes can operate maliciously to manipulate the assets within the blockchain. On the other hand, private blockchains allow only authorized nodes to participate in the network and exchange digital assets [18,19].

Although blockchain’s popularity started in 2009 in the financial sector with the use of cryptocurrencies (eg, Bitcoin), it has grown and expanded to different industries, including health care, legal, security, and government [20,21]. In health care, various proof of concepts and pilots are in progress to innovate processes and address longstanding problems in handling data [22]. As a distributed digital ledger, blockchain is believed to have the potential to mitigate some of the intractable issues in health information exchange and data management [23]. With the growing interest and development in this field, both from the private industry and government, there is a need to better understand how blockchain technologies could support opioid-related health outcome studies.

In this paper, we explore the different applications of blockchain and how it may be strategically deployed to facilitate opioid-related research. This paper can serve as a helpful resource for researchers, health IT innovators, and other stakeholders in exploring blockchain technologies to address data challenges and data infrastructure gaps. This paper also aims to stimulate discussions on potential implementation challenges and encourage more research to generate evidence on blockchain’s applicability in health care research.

## Methods

This paper is informed by two primary data collection activities: (1) a search and review of peer-reviewed and gray literature and (2) a technical expert panel (TEP).

### Literature Review

A literature review was conducted to identify challenges in conducting opioid-related research and to understand the potential uses of blockchain to address those challenges, including its limitations and other implementation considerations. Given that the application of blockchain to health care is an emerging field, peer-reviewed and gray literature was assessed in this paper. Search strings used included terms related to longitudinal health records, data sharing, and research applications for blockchain, as well as direct references to blockchain and opioids. Searches were conducted using PubMed, Google Scholar, and Google search engine. Supplemental literature was obtained from three additional sources: (1) the TEP and a subject matter expert advising the team, (2) government papers and reports [16], and (3) white papers from the industry and academia [24]. We conducted a title and abstract review of the 458 search results, followed by a full-text review that yielded 104 relevant articles.

### TEP Engagement

As the use of blockchain in health care is new and emerging, the literature contains limited technical information on the technologies and minimal assessment of their feasibility. As such, 3 experts were recruited from the field of health IT, blockchain technology development, and opioids research to offer an *on-the-ground* perspective. In particular, we discussed research challenges associated with opioid outcomes research, how blockchain applications relate to opioid research in particular, and challenges and recommendations for using blockchain in opioid research.

## Challenges in Opioid Research

The number of opioid-relevant data sources, their diversity, and natural heterogeneity creates challenges for opioid researchers. To assess the effectiveness of interventions and programs directed to help address the crisis, researchers and clinicians need data that are accessible, high quality, robust, and timely [25]. They also need improved tools and services that allow researchers to better manage opioid-related data, as well as improve the efficiency of data integration throughout the research life cycle [6]. The challenges identified in the literature also highlight the need to harmonize and link data sources for analysis [26] and overcome systemic barriers to interoperability [27]. Opioid use disorder patient data tend to be scattered across institutions and service points (eg, criminal justice, health care, and substance abuse treatment systems) that are involved in providing care, interventions, and assistance. As these institutions have information systems that are not designed to interoperate with other systems, they create barriers to effective care coordination for clinicians and longitudinal data access for researchers [25].

Another unique challenge in opioid research is the 42 Code of Federal Regulations (CFR) restriction on the disclosure and use of records of patients with substance use disorder (SUD), which are maintained in connection with a part 2 program. Part 2 programs are federally assisted programs for individuals, entities other than a general medical facility, or an identified unit within a general medical facility that *holds itself out* as providing SUD diagnosis, treatment, or referral for treatment [28]. As patients with SUD can potentially face stigma and discrimination, their data are protected by stringent privacy policies [29]. Although privacy policies such as the Health Insurance Portability and Accountability Act of 1996 and 42 CFR Part 2 encourage patients to seek care without thinking of potential negative consequences, they inhibit communication among providers and sharing of information through health information exchange unless very narrow circumstances are met and patient consent for data sharing is obtained [30]. As a result, researchers have limited access to real-world data [31] that are essential for tracking patient trajectories, conducting risk prediction, and outcome analysis.

Textbox 1 highlights the challenges identified in the literature review. The textbox also provides more context and descriptions of the challenges.

**List of challenges in conducting opioid research.**

**Recruitment and Retention of Study Participants**

- Patient skepticism about research and the health care system and concern about confidentiality and privacy [32] often result in lower consent rates.
- Difficulty and cost of collecting longitudinal health data about substance use disorder, particularly because study attrition rates are often high.

**Data Integrity, Accuracy, or Completeness**

- Lack of completeness in electronic health records and other data sources due to absence in alignment between workflow and documentation, user error, and use of nonstandardized free-text fields that cannot easily be converted into analyzable data.
- If data are not entered into the electronic health record, the data are not available for research (eg, patient pain agreements that outline opioid use disorder preventive actions and paper prescriptions) [8].
- Underreporting of opioid use and deaths due to challenges associated with collecting vital statistics and hospital data [33]:
  Death certificates do not always specify drugs that contributed to death, causing researchers to underestimate opioid overdose deaths [34].
  Neonatal abstinence syndrome surveillance relies primarily on hospital discharge data, which may not capture cases of opioid use disorder diagnosed during other points in pregnancy [35,36].
- Limitations of self-reported opioid use in nationally representative surveys stemming from participants’ lack of knowledge on opioid misuse, overly broad questions (eg, on overall use, rather than specific opioids), and lack of awareness about exposure to adulterated drugs [37].

**Timeliness of Data**

- Reporting to prescription drug monitoring programs can vary across states, between 5 minutes and 8 days [38]. The latest report from the National Vital Statistics System on drug overdose deaths and the current National Survey on Drug Use and Health data are still from 2018 [3,39].
- Some Medicare data files have a lag time of 2-4 years [40].

**The Need for Linked Data**

- Opioid data exist in silos across health systems [41]: first responder organizations, medical examiners or coroners, law enforcement entities, criminal justice entities, treatment providers, and other stakeholders.
- Differences in unique respondent identifiers between data sets can make it difficult to match respondents’ data.

**Data Security and Privacy**

- Substance use disorder or opioid use disorder data are subject to additional regulations as “sensitive protected health information,” including opt-in consent policies (42 Code of Federal Regulation Part 2) and Health Insurance Portability and Accountability Act.
- Disclosure of information that would identify opioid use disorder requires written consent, which limits the data available for opioid research [42].

**Data Sharing and Interoperability**

- Data sharing agreements among public health departments, providers, health systems, and federal agencies are complex and time intensive to create [43].
- The high cost of data exchange is a barrier [44].
- Lack of interoperable electronic health records prevents information sharing on prescriptions, and prescription drug monitoring programs or electronic health record integration has not been widely implemented.
- Prescription drug monitoring programs are operated individually by state governments, and requirements around who is able to or who is required to access them vary widely [38].

**Data Collection Gaps at the Point of Care and Secondary Use of Clinical Data**

- Adoption and awareness of opioid prescribing guidelines vary widely [8]
  Providers may not consider nonopioid alternatives for pain management.
  Providers may not prescribe appropriate opioid types, doses, and quantities or durations tailored to the patients’ specific types of pain (eg, acute vs chronic).

## Blockchain Applications

### Overview

There is growing interest among health IT innovators in the application of blockchain in health care. Specifically, blockchain is used to address issues related to data availability, interoperability, and privacy and security, as they relate to care management and health research [45-47].

The value of blockchain in research can be demonstrated through its potential in improving health research at multiple levels by improving data quality, reducing cost, decreasing administrative delays by fast-tracking vendor proposal review and contract purchasing, and accelerating the time needed to translate research to practice [48]. When applied to specific points in the research lifecycle, blockchain could potentially increase access to research studies by making more publications available through its network, streamline processes for securing funding [49], and improve transparency to prevent tampering and misreporting of data findings [50].

Although blockchain cannot be characterized as a panacea for the many challenges in opioid research, the results of the literature review and inputs from the TEP indicate that it may be strategically deployed to solve several major challenges. The 5 applications that emerged consistently in the literature as promising areas for applying blockchain are summarized in Table 1 with details about the problem area, ways blockchain could potentially address the problem, and a real-world example.

**Table 1:** Blockchain applications to address opioid research challenges.

Blockchain application themes	Needs or problem areas identified	How blockchain can be used to address opioid research challenges	Real-world example
Clinical trials and pharmaceutical research	Management of and transparency in reporting clinical trials and consent management	Creating a trusted record to support clinical trials with public data and findings and facilitating data movement via secure sharing and consent	Simulation of a clinical trial study (using actual raw data) on the efficacy and safety of omalizumab (asthma and chronic idiopathic urticaria drug) using a blockchain-based system to test the resilience of the data to tampering and improve traceability [51].
Incentivizing data donation and behavior change	Sharing of data for research and healthy behavior change	Use of cryptocurrency to distribute incentives that target data donation and adoption of healthy behavior	A secure and transparent distributed personal data marketplace (using blockchain) that allows users to sell their biomedical data and customers to buy data for research and analysis using cryptocurrency [52].
Secure exchange and management of electronic opioid prescriptions	Inaccurate prescription data, multiple active opioid prescriptions, and the need for better tools and systems for monitoring opioid prescriptions	Reducing fraud in e-prescribing and improving timeliness and ease of data reporting and surveillance	A solution that uses blockchain to track specific prescriptions using a machine-readable code from the time a drug was prescribed to distribution by the pharmacy. The code, which serves as the unique identifier, is associated with the prescription information, thereby augmenting PDMP^a^ data and allowing pharmacists to verify its accuracy and eligibility to be filled [53].
Supply chain management	Theft or diversion of drugs, the introduction of counterfeit medicines, and contamination of drugs during production and distribution	Improving drug traceability	A pilot project uses blockchain technology to assist the US Food and Drug Administration and members of the pharmaceutical distribution supply chain in the development of an electronic, interoperable system to identify and trace certain prescription drugs as they are distributed within the United States [54].
Secondary use of clinical data	Patient data stored in different information systems, interoperability, availability of longitudinal data	Increasing researchers access to longitudinal and population-level outcome data that support research and near real-time public health surveillance	A blockchain-based information management system that allows secure access and sharing of patient records from one EHR^b^ to another [55].

^a^PDMP: prescription drug monitoring program.

^b^EHR: electronic health record.

### Clinical Trials and Pharmaceutical Research

Among the different applications of blockchain in health care, its use in clinical trials and pharmaceutical research is cited as among the most promising [56-59]. Research is important for developing the tools and understanding to identify new treatment options, identify ways to prevent adverse outcomes, and identify other viable opioid alternatives to manage pain [60,61]. Furthermore, trust in the validity of research data and analysis is critical for translating clinical trial results to quality clinical care. Consent collection and management is one area where blockchain can be used to support research. Using *smart contracts*, a simple computer program that is used in blockchain to digitally facilitate, verify, and enforce contracts [62], can help researchers capture all aspects of data that might be subject to manipulation including trial registration, protocol, subject registration, and clinical measurement. This technology can be used to record patient consent to participate and allow consent to be audited to ensure adherence to recruitment guidelines [63].

Blockchain can also support clinical trial management and reporting. Incomplete and inaccurate reporting of clinical trial data [64,65] can lead to problems for regulators such as the Food and Drug Administration in auditing data, real-time oversight, and adverse event reporting [23,51]. Compliance with 21 CFR Part 11 for late stage preclinical and clinical research can be cost-prohibitive for companies, which might compromise data integrity; however, distributed clinical trial management and trial data via blockchain can create an immutable audit trail that allows users to ensure that the results have not been tampered with [64,66,67]. For phase IV clinical trials that look into drug or device safety over time, regulators could use blockchain smart contracts to automatically query clinical trial sites for adverse events [23].

Blockchain can allow researchers to maintain ownership of study data while publishing results in real time. This could encourage faster dissemination of results and potentially seed collaboration with other researchers working on the same topic [68]. Specific to opioids, blockchain “could help the research and development of opiate alternatives by laying the groundwork for a decentralized database of [laboratory] test results with free access to this data” [59], which could lead to more cost-efficient drug development [67].

Several prototypes use blockchain technology as a data platform, in addition to existing distributed clinical trial infrastructures. One example is a prototype developed by a university that enables access to research trial data abstracted from EHRs. It uses blockchain technologies such as cryptography and smart contracts to record patient consent and to track distributed researcher queries from trial data repositories stored *off-chain* [67]. A proof of concept was developed, which makes data collection within the trial life cycle immutable yet traceable and potentially more trustworthy [51]. Another blockchain-based platform was developed to assist pharmaceutical and biotechnology industries in simplifying recording processes and ensuring data integrity and fidelity in all phases of the research process [63,69].

### Incentivizing Data Donation and Behavior Change

Blockchain technologies can encourage patients to share their medical information with researchers, both through secure sharing and incentives. These data increase researchers’ access to longitudinal data, which then support better outcome research to study the opioid crisis [70,71]. The use of blockchain can reward users through cryptocurrency to participate in networks [72]. It could also shift data stewardship from centralized authorities (eg, the National Institutes of Health, research networks, or academic research centers) so that patients and researchers can manage their data access rights [73].

One example is a platform for storing patient data that leverages artificial intelligence and blockchain technology to support a marketplace for individuals and biobanks to store, manage, and control access to genomic and other health data [52]. A number of companies [74,75] also manage DNA marketplaces where individuals can share DNA data in exchange for cryptocurrency, which are then purchased by researchers.

In addition to incentivizing data sharing, blockchain technologies can drive users to follow health recommendations. Examples of existing platforms target the adoption of wellness activities [76,77] and reduction of doctor appointment no-shows [78]. Another platform rewards patients with cancer through cryptocurrency to report side effects, medication adherence, and healthy lifestyle choices. Oncologists can also receive rewards to create content to help monitor the patient’s inputs [79].

The idea of providing nonmonetary incentives in the area of substance abuse is not new, and many studies have already demonstrated positive outcomes [80-82]. Although the identified examples are not opioid-specific, they illustrate the blockchain’s potential in this use case. Researchers can implement blockchain-enabled technology to incentivize participation in studies, attend follow-up sessions, submit patient-reported measures, and encourage adherence to medication and treatment.

### Secure Exchange and Management of Electronic Opioid Prescriptions

Moving from paper to electronic prescribing reduces (but does not eliminate) prescription theft and forgery and facilitates tracking of prescription histories for prescribers, pharmacists, and patients [8]. When securely stored on a blockchain, e-prescriptions can be made tamperproof, which is further secured by monitoring for potential misuse [83].

Blockchain-enabled systems are believed to be beneficial for the real-time capture and verification of prescriptions. One company developed a platform that uses a process to ensure e-prescription fidelity using blockchain. First, every new prescription is assigned a unique identifier in the form of a machine-readable symbol that is associated with a block of prescription details (eg, drug, dosage, anonymized patient identifier, and timestamp). Second, pharmacists can scan the code, verify that the data block matches the prescription details, and document that it has been filled. The blocks are stored in multiple places as part of the distributed ledger system, encrypted during transmission, and only accessible with the correct cryptographic key, and therefore more trustworthy [84].

Leveraging electronic prescription technology, PDMPs are intended to capture and disseminate real-time information about opioid prescribing practices and prevent *doctor and pharmacy shopping* in which a patient seeks the same prescription from multiple providers or attempts to fill prescriptions at multiple pharmacies to decrease fill denial [85]. Having access to timely PDMP data provides researchers the opportunity to analyze prescribing patterns, which are central in combatting opioid misuse and addiction. Unfortunately, PDMP reporting intervals vary widely, ranging from 5 minutes to 8 days [36]. A blockchain-enabled monitoring system would allow real-time verification of previous prescriptions by doctors as they consider prescribing new opioids, followed by real-time reporting of new prescriptions, and real-time verification by pharmacists filling that prescription. Reducing the time and friction of data transfer also reduces costs and can be deployed nationwide instead of state by state [86].

### Supply Chain Management

Addressing supply chain issues such as theft or diversion, the introduction of counterfeit medicines, and contamination of medicines during manufacturing, storage, or distribution [87] by ensuring the provenance and authenticity of the drugs are crucial to patient safety. Blockchain can help with 2 traceability issues. First, it allows companies to track their products down the supply chain, creating a circuit that is secure and difficult to penetrate by counterfeit products. Second, it allows stakeholders, especially laboratories, to identify the exact location of their drugs in case of a problem [88].

Several studies have proposed applying blockchain to supply chain information exchange and data storage [87,89-91]. Transaction information regulated by the 2013 Drug Supply Chain Security Act [89], that is, product information, transaction history, and ownership, can be stored and managed on a blockchain [87]. This allows network participants to track and validate a drug pill by pill [88] from manufacturing to dispensing, detecting anomalies and identifying missing drug products and unauthorized data insertions [92]. The private industry has shown great interest in this use case and has developed several working platforms and proof-of-concept designs exploring the feasibility of blockchain in addressing supply chain issues [89,93,94]. Another pilot project focused on applying blockchain-based technology to supply chain management by identifying unused oral cancer drugs and giving them to patients who cannot afford them [95].

Specific to opioids, the US government and private companies are testing the use of blockchain-based supply chains to help improve the security of prescription drug supply and distribution and to allow real-time monitoring of pharmaceutical products [96]. Various efforts are also being implemented to track dispensing [97] and expand access [98] to naloxone, a drug used to counteract opioid overdoses. Although policies allowing naloxone dispensing through standing order have shown a significant reduction in opioid-related deaths [99-101], this could create information gaps regarding its distribution and use. As naloxone is distributed to first aid responders and laypersons, the recording of its use may not always be consistent. Having access to this information could help public health officials and researchers identify trends in opioid use using naloxone as a marker for opioid overdose and assess the impact of local policies related to naloxone distribution efforts [102,103].

### Secondary Use of Clinical Data

A comprehensive and accurate view of a patient’s trajectory over time, across providers, and across health care and non–health care settings is crucial to the ability to precisely answer research questions and conduct near real-time public health surveillance. However, patient health data are often collected and stored in disparate information systems (eg, emergency department registries and first responder data systems), greatly reducing researchers’ access to longitudinal records for real-world evidence generation for opioid-related treatment [55,104] and public health surveillance [19]. Accessing data from disparate health information systems requires high overhead costs, and systems often lack basic computer security protocols to authenticate patient data.

Aside from access concerns, dealing with substance use data is unique because it requires special and careful handling. Privacy and security are critically important, given that SUD-like opioid misuse remains highly stigmatized and is therefore classified as *sensitive protected health information*. This means that they require extra protection and consent requirements under 42 CFR Part 2 [105], creating obvious barriers for opioid researchers [40] and information exchange [106]. Data from nonsubstance abuse treatment providers only provide part of the picture of a patient’s trajectory. SUD and associated SUD treatment data are crucial to an understanding of patient outcomes in relation to treatment settings (eg, detox and residential) and treatment types (eg, medication-assisted treatment). Blockchain can provide structure and security for improved data sharing by creating a concurrent, distributed, redundant, and secure system that facilitates record linkage and improves interoperability between data sharing partners [107] for research and surveillance use cases.

An example of how blockchain technology can be implemented to facilitate record linkage is a platform [55] that was developed to gather and link information from disparate patient records without central data storage. It allows authorized users to upload encrypted clinical summaries for cross-system sharing and to easily search and retrieve patient health information that has been shared across systems. Government health agencies are also exploring ways to further maximize the potential of information in EHRs and how blockchain can be used for public health surveillance [108]. Blockchain is seen as a technology that could complement the public health’s complicated peer-to-peer model for data sharing “to more efficiently manage data during a crisis or to better track opioid abuse” [109].

## Discussion

### Overview

The abovementioned applications demonstrate the potential of blockchain to support opioid research. However, blockchain is a relatively new technology in health care, necessitating a critical assessment and testing of its suitability for the opioid research challenges identified in this paper. It is also recognized that blockchain is not only and may not be the best solution for each of the identified gaps. On the basis of the literature, the application of blockchain in clinical trials and pharmaceutical research is ready for more real-world implementation. Other applications could provide evidence of data fidelity and provenance to improve the broad and timely sharing of clinical trial data and accelerate the pace of research [63].

Input from the TEP regarding blockchain applications with the most potential impact on opioid research include increasing access to longitudinal and population-level outcome data for research and surveillance [55] and supply chain management [57,88], with an emphasis on monitoring administration of opioid overdose reversal medication. The TEP also highlighted areas where blockchain could potentially support the administrative side of health care research: grant proposal processing and review; financial distribution of research funds and longitudinal tracking of dollars to demonstrate return on investment; regulatory tracking and auditing of research that lower admin cost while ensuring compliance; and facilitating more rapid dissemination of findings.

### Challenges and Limitations

#### Overview

Blockchain applications in health care are still in their infancy. There is a need to further study and test the true feasibility for health care applications in general and in areas that require a high degree of privacy and confidentiality protection (eg, SUD). Near-term opportunities to support growth in the blockchain market should focus on real-world implementation to assess challenges and limitations.

#### Technical Challenges

There are several technical challenges in using blockchain for health care research. First, existing data infrastructures may require new mechanisms to interface with blockchain. For example, collecting and managing data with blockchain solutions may require technical upgrades to existing systems. Depending on the data sources (eg, EHRs, distributed research network data marts, clinical trial registries, and PDMPs), different technical solutions may be required at varying costs and complexities [110]. Relatedly, the TEP identified a need for discussion on internet requirements and capabilities for running these new blockchain applications.

Second, the lack of metadata standards for information stored in the blockchain may challenge interoperability. This includes a lack of standards around smart contracts, which are needed before the blockchain can be applied consistently [63].

Third, blockchain does not address all existing challenges in data interoperability and validity. There is wide variability in the standardization of electronic health information, which is not solved by blockchain. Data validity concerns are also not fully resolved by blockchain because data stored off-chain can still be manipulated before being added to the ledger [66].

Fourth, not all use cases have a clear business model to incent implementation and participation. Despite the potential for many of these use cases, they all require investments in infrastructure and incentives for adoption and use [111], and for networks, it is necessary to determine how to distribute costs.

Finally, scalability with regard to processing power has been identified as a key challenge to implementation [112]. The use of blockchain to store vast amounts of data (eg, millions of patient records, multi-institutional data, and global research records) can incur equally vast storage costs. Blockchain networks have defined data size limits that may be quickly exceeded, depending on the use case [113].

#### Policy Challenges

A complicated legal and regulatory framework governs the use and disclosure of patient health information, with additional federal and state laws governing specially protected health information, such as substance use (eg, 42 CFR Part 2, section 3221 of the Coronavirus Aid, Relief, and Economic Security Act) and genetic data (eg, Genetic Information Nondiscrimination Act and the US National Institutes of Health Genomic Data Sharing policy). Some state and international laws have requirements regarding data destruction upon revocation of patient consent [114,115]. This has prompted some organizations to exclude specially protected health information from their sharable records. Understanding how blockchain implementations can link and integrate SUD treatment records with other health care data while managing confidentiality requirements is critical to the application of blockchain to opioid research and treatment. Examining blockchain implementations in Europe may provide some early lessons learned regarding how implementers manage blockchain immutability while complying with the General Data Protection Regulation.

One of the key characteristics of blockchain is that it promotes trust in transactions and records—a significant attribute given patients’ expectations of privacy and confidentiality. Ambiguities in current and future policies governing cryptocurrency may limit its potential and challenge users to comply with legal requirements (eg, if cryptocurrency or blockchain tokens are classified as security, they will become subject to Securities and Exchange Commission rules) [62]. Federal regulators should consider their role in ensuring trust in the larger clinical trial ecosystem and other data donation initiatives by encouraging the private industry to adopt strong privacy and confidentiality requirements. For example, the CARIN Alliance has developed a trust framework and voluntary code of conduct for stakeholders and organizations entrusted with personally identifiable information [116].

The industry also needs data governance rules for which entities can write data to an official chain. For example, blockchain can reduce the burden of credentialing for providers by enabling providers to self-assert rather than requiring an intermediary such as a medical board to issue the claim on behalf of the provider [86,117]. In some instances, this may require changes to existing laws regarding the use of digital signatures, which support writing information in a chain.

In terms of policies related to blockchain, most regulatory discussions are happening at the federal agency level around its use for cryptocurrency. This is despite the recognition of blockchain applications in other areas. In 2019, there were 27 state bills and resolutions relating to blockchain, which have been enacted or adopted. These resolutions tackle more applications outside of cryptocurrency, such as examining blockchain’s use for elections (Connecticut and New York), state administrative transactions (Connecticut), and health care use cases (Virginia) [118,119].

### Potential Opportunities

Solving these technical and policy challenges requires a coordinated approach between the private and public industries, as illustrated by the real-world examples presented herein. To further explore blockchain to opioid-related research, the TEP encouraged work in the following areas:

1. There is a general lack of education and understanding of blockchain, which challenges its application in research. Raising awareness among key research stakeholders will increase knowledge and build competencies, priming the research community for implementation. Given the technical and policy challenges, researchers should be encouraged to use industry guidance to assess the feasibility of blockchain applications. The National Institute of Standards and Technology may offer one such tool to focus on researchers and developers working to identify near-term opportunities that are fit for blockchain [16].
2. There is a lack of standards for smart contracts. Given that many blockchain applications rely on smart contracts, developing standards and policies can improve interoperability within a network [63].
3. The legal and regulatory environment for the use and disclosure of substance use treatment information poses challenges for data sharing [29]. Studies related to the technical solutions for implementing blockchain in this complex ecosystem (considering various policy and privacy requirements) are needed to realize the potential of integrating SUD treatment records with other health care data to track patient outcomes over time.

### Conclusions

Researchers are currently faced with a number of challenges with access to and use of opioid-related data. Aside from the need for high-quality, accessible, robust, and timely data, opioid researchers are also confronted by siloed information systems and the privacy requirements for SUD data. Considering the features and capabilities of blockchain and its current application in other industries, it has the potential to act as a facilitator to address these challenges by offering a more efficient, secure, and privacy-preserving solution to the research process, data management, and data exchange. Among the 5 primary applications that we identified, its use in clinical trial research, supply chain management, and secondary use of data for research and public health surveillance had the most evidence for implementation opportunities and potential for the effectiveness of blockchain. Although these blockchain applications present great potential, future work should understand and address concerns related to standards, infrastructure, scalability, implementation cost, sustainability, and governance. Policy concerns related to balancing the need to create high-fidelity data that also protect patient privacy and patient autonomy in revoking consent to use their data for research and treatment should also be addressed. Discussion and evidence generation efforts should focus on addressing these challenges to evaluate the feasibility and at the same time maximize the potential of blockchain technology.

## Abbreviations

CFR: Code of Federal Regulation
EHR: electronic health record
IT: information technology
PDMP: prescription drug monitoring program
SUD: substance use disorder
TEP: technical expert panel
